# Shared Death Anxiety: Obsessive-Compulsive Disorder and Advanced Paternal Age

**DOI:** 10.7759/cureus.77547

**Published:** 2025-01-16

**Authors:** Tiago Aguiar Soares, Joana Coelho Santos, Daniela Couto

**Affiliations:** 1 Department of Child and Adolescent Psychiatry, Local Health Unit of Western Lisbon, Lisbon, PRT

**Keywords:** advanced paternal age, child and adolescent psychiatry, death anxiety, elderly parent, family dynamics, obsessive-compulsive symptoms, parent-child relationship

## Abstract

Obsessive-compulsive disorder (OCD) often emerges during childhood, presenting as distressing obsessions and compulsions that interfere with daily functioning. Advanced paternal age (APA) introduces unique psychosocial dynamics within families, potentially exacerbating stress, anxiety, and intergenerational emotional challenges. This case report examines the presentation of OCD in a 10-year-old boy, characterized by pervasive death anxiety shared with his 83-year-old father, whose advanced age intensifies existential concerns. The report delves into the complex interplay between APA, the child’s obsessive-compulsive symptoms, and family dynamics marked by a dichotomy of the mother’s enmeshment and the father’s emotional distancing. Cultural considerations also play a critical role, as the mother’s Filipino heritage introduces unique values and practices that influence caregiving dynamics and coping mechanisms. Therapeutic strategies tailored to this multifaceted context are explored, focusing on alleviating OCD symptoms, strengthening family cohesion, fostering open dialogue about existential fears, and addressing culturally sensitive aspects of care. This case underscores the critical need for personalized and culturally attuned interventions that address the psychosocial challenges associated with APA, highlighting their profound impact on pediatric mental health outcomes.

## Introduction

Obsessive-compulsive disorder (OCD) is a neuropsychiatric condition affecting approximately 1%-3% of children, often manifesting between the ages of 7 and 12 [1,2]. Characterized by debilitating intrusive, distressing thoughts (obsessions) and/or repetitive behaviors (compulsions), OCD disrupts various aspects of a child’s life, including academic performance, social interactions, and familial relationships [1]. Early detection and treatment are critical to mitigating these adverse outcomes [2]. Despite advancements in understanding the pathophysiology and treatment of childhood OCD, the interplay between genetic, neurobiological, and psychosocial factors continues to challenge clinicians [1,2].

Advanced paternal age (APA), defined as fatherhood at or after the age of 40, is a rising demographic phenomenon associated with increased risks for neurodevelopmental and psychiatric disorders, including autism spectrum disorder, schizophrenia, and OCD [3,4]. The biological underpinnings of these risks include de novo genetic mutations, epigenetic alterations, and age-related changes in sperm quality [5,6]. Beyond genetic factors, APA introduces unique psychosocial dynamics within families, influencing parenting styles, attachment patterns, and children’s emotional development [7].

In APA families, existential concerns such as death anxiety can profoundly affect the child-parent relationship. Death anxiety, a pervasive fear of mortality, may manifest in children as obsessive thoughts or compulsive rituals aimed at mitigating perceived threats [8]. These rituals often receive unintentional reinforcement through family accommodations, such as participating in the child’s rituals or avoiding discussions about distressing topics. Such dynamics exacerbate OCD symptoms and hinder adaptive coping mechanisms [9].

Cultural factors further shape family dynamics and caregiving behaviors. In Filipino culture, close mother-child relationships are commonly observed, with practices such as co-sleeping and heightened maternal involvement playing a prominent role [10]. These culturally rooted behaviors can foster a strong sense of security in children but may also reinforce dependency and enmeshment in families where psychological stressors, such as OCD and death anxiety, are present [8-10]. Understanding these cultural nuances is critical for designing effective, culturally sensitive interventions that address the unique needs of diverse families [10].

This case report explores the intersection of APA, death anxiety, and OCD in a 10-year-old boy. It highlights the complex interplay of genetic predisposition, family dynamics, and psychosocial stressors, emphasizing the importance of holistic, culturally sensitive, family-based therapeutic interventions.

## Case presentation

The patient is a 10-year-old boy living with his 83-year-old Portuguese father and 52-year-old Filipino mother. He has a 21-year-old brother. Academically, the child excels, participates actively in extracurricular activities, and enjoys drawing and playing board games. He identifies as Catholic and lives in a spacious home that regularly hosts members of the local Filipino community.

He initially presented to the emergency room (ER) of child and adolescent psychiatry at the end of 2021 with a pattern of ego-dystonic obsessive-compulsive behaviors, including cleaning, checking, and repetitive rituals. These behaviors were frequent, occurring daily, with each episode lasting at least five minutes and often followed by another compulsion. At home, he appeared very nervous and agitated, walking around and repeating his rituals to achieve temporary relief from his anxiety. This anxiety seemed to be both related to his compulsions and a profound fear of death, as noted in his ER presentation and subsequent clinical interviews. In the ER, he cried, expressing that he urgently needed to see a doctor because he felt he was dying. Although he recognized feeling like this while at home, he did not share it with his parents.

The onset of symptoms could not be precisely determined, but the family estimated that the behaviors began in early summer 2021, suggesting a duration of approximately half a year before the ER visit. Several potential triggers were identified as possible contributors to the escalating anxiety, including recent exposure to an age-inappropriate violent video game based on "Squid Game," which he played for 2-3 hours per day over several weeks during the summer. His mother had already warned him against playing such games, expressing her disapproval, but he struggled to stop. He also underwent two minimally invasive surgeries: a circumcision with a minor complication in July 2021 and an uncomplicated appendectomy in October 2021.

Physical and neurological examinations conducted at presentation were unremarkable. Laboratory investigations, including a complete blood count and metabolic panel, were within normal limits.

The compulsive behaviors manifested across several domains (Table 1). Cleaning behaviors were confined to his bed area during the evening and night. Initially, he explained this as being due to visible dust, but later he described these behaviors as a way to ensure no monsters were under his bed. Checking behaviors included repeatedly looking under the bed and behind his room’s door, as well as compulsively checking and rearranging his father’s slippers by the side of his father's own bed (in a separate room). Repetitive behaviors included switching the lights on and off four times and occasionally touching random objects while walking through the house, such as walls, pictures, and other decorations.

**Table 1:** Overview of the child's compulsive behaviors

Category	Description
Cleaning	Cleans around his bed only in the evening and night. Initially explained as a response to visible dust, later linked to the checking behavior to ensure no monsters were under the bed.
Checking	Under the bed and behind the room’s door checking for monsters. Checks and rearranges the position of his father’s slippers next to his father's bed.
Repetition	Switches the lights on and off four times. Occasionally touches random objects while walking around the house (wall, pictures, decoration, etc.).

In the ER, he was prescribed risperidone titrated to 0.75 mg once daily at night and referred to the outpatient child and adolescent psychiatry clinic. The mother was advised to ensure the child had no access to violent media.

The patient was seen at the outpatient child and adolescent psychiatry clinic two months later, accompanied by his parents. His mother had discontinued the risperidone after three weeks due to his complaints of unbearable headaches and dizziness. The obsessive-compulsive behavior persisted. The patient expressed that his behaviors were driven by obsessions focused on death, particularly his own and his father’s. He denied having intrusive images, cognitive compulsions, or aggressive thoughts and reported no changes in his sleep, feeding, or elimination patterns.

A mental state examination revealed a boy with an apparent age congruent with his chronological age. He was clean, well-groomed, and appropriately invested. He appeared vigilant and oriented in all four dimensions. Initially inhibited, he became more engaged and accessible as the consultation progressed. His eye contact was directed, and he was cooperative, though slightly uneasy when discussing personal matters. His mood was euthymic with an anxious undertone, particularly when discussing his sleep habits or the fear of his parents’ death. His affect was broad, mobilizable, and congruent with his speech. The speech was spontaneous, fluent, and coherent. There were no disturbances in the form or course of his thought processes, and he denied any sensory-perceptual disturbances or alterations in his self-experience. He demonstrated insight into his condition and expressed a desire for help. His introspection appeared appropriate for his developmental stage, and his cognitive abilities were subjectively assessed as good. No psychometric testing was conducted.

The patient willingly engaged in drawing during the consultation, producing a detailed depiction of a house with an unusual architectural style. The house had three stories, a cross-shaped window (possibly representing an elevator), large windows, a garden with a pool, and a telescope on the roof. Outside the house boundaries, in the corner of the page, he drew minimally invested human figures representing his family. When asked about his future, he stated he aspired to become an architect or engineer.

The family dynamic revealed a fusional relationship between the child and his mother, who often slept in his room and closely supervised him, rarely leaving him alone. In contrast, the father was emotionally distant, citing his advanced age and declining health as limiting factors although he had no current or past significant health issues. He exhibited a sub-depressive mood, making indirect comments about his own mortality and expressing regret about not being able to accompany his son’s growth. The subject of death was completely avoided within the family and created visible anxiety in all elements when addressed.

The patient’s developmental history was notable for a pregnancy that was unplanned but desired. Both pregnancy and delivery at 38 weeks were uneventful, with no prenatal, perinatal, or postnatal complications. The child weighed 3,420 g at birth and was named by his older brother after his best friend. The mother served as the primary caregiver during infancy, with occasional support from the father. She described her mood during this period as positive, feeling secure in her role. There were no separations from caregivers. The patient’s brother has had an overall close and supportive relationship; they both trained in karate at the same school. During their childhood, they shared a room. In the last four years, however, the brother joined the navy and now returns home intermittently, occasionally sleeping in the same room as the patient. The patient was breastfed until the age of three and has co-slept with his mother until the present day, which is common in Filipino culture. Currently, his room contains two beds: one used by his mother (and occasionally by his brother) and the other by the patient. The father has his own room and usually goes to bed earlier.

The child exhibited a calm temperament and met developmental milestones within the expected range, including walking at 13 months, first words at 16 months, and achieving day and night sphincter control by age 2. He transitioned to preschool at age 3 without difficulty adapting or signs of separation anxiety. He suffered bullying during the first three years of primary school due to his weight. Currently, teachers and peers describe him as friendly, cooperative, and fully engaged, with no reported difficulties in forming or maintaining interpersonal relationships. He has active participation in various activities with peers in and out of school. He also demonstrates a good capacity to regulate frustration, accepting limits easily and without oppositive or argumentative behavior. 

Regarding the family’s medical and psychiatric history, the father shows signs of a current depressive tendency but denies experiencing previous depressive episodes and has not sought formal evaluation or treatment. Born into a very poor family with numerous siblings, he spent many decades working abroad before returning to Portugal. He has a history of coronary artery disease with left ventricular hypertrophy and diastolic dysfunction, aortic insufficiency, and hypertension. There is no known family history of major hereditary illnesses. The mother displays mild anxiety traits but manages her daily responsibilities effectively. There is an aunt reportedly experiencing auditory and visual perceptions that others do not share. She is described as functional and appears to exhibit traits suggestive of a schizotypal personality, although she has never undergone formal evaluation. No other family history of psychopathology is known.

​​​​The patient’s physical health history included a diagnosis of obesity (BMI ≥ 30) and a slightly asymmetrical tricuspid aortic valve with minimal regurgitation but no stenosis, diagnosed in 2017; no medication was indicated. He underwent a circumcision in October 2021, which was complicated by an infection and mild swelling, and a laparoscopic appendectomy in July 2021 without complications. There is also a history of minor allergic reactions to bee stings and kiwi.

Management and treatment

The management of this case involved a multidisciplinary and culturally sensitive approach, addressing the child’s psychological, behavioral, and family needs. Follow-up consultations were scheduled every three weeks to monitor progress and refine the treatment plan.

Psychological treatment focused on cognitive behavioral therapy (CBT) with exposure and response prevention (ERP) to target compulsive rituals. These sessions happened for six months and took around 45 minutes each: weekly for the first month, biweekly for two months, and then monthly for the final three months. Cognitive restructuring was employed to address the child’s death-related obsessions. To help the family process existential concerns, therapy encouraged open communication about mortality, particularly the child’s perception of his father’s advanced age and potential death. The family was advised to watch and discuss age-appropriate animated films (*Disney's Coco, The Lion King, Big Hero 6, and Up*) that subtly address themes of loss and resilience. Afterward, the emotions depicted in the films were explored, with parallels drawn to the child's own situation.

The family’s dynamics were a critical focus of the intervention. Therapy aims to strengthen the bond between the father and the child through shared activities while reducing the fusion between the mother and the child to encourage the child’s independence. Psychoeducation was provided to both parents, addressing the implications of APA and the role of expressed death anxiety in exacerbating OCD symptoms. The active involvement of the local Filipino community in the family's home provided an opportunity to explore its system of beliefs and how these influenced the family’s dynamics. Efforts were made to ensure that these cultural and spiritual practices served as a source of emotional support for the child, rather than contributing to heightened anxiety or reinforcing maladaptive behaviors. This culturally attuned approach allowed for a personalized intervention that aligned with the family’s values while fostering a stabilizing and supportive environment for the child.

Behavioral strategies included promoting independent behaviors, such as sleeping alone, supported by positive reinforcement techniques like behavior charts. Symbolic objects, such as a “magic sword” placed near the bed and a “monster spray,” were introduced to alleviate nighttime fears related to teratophobia.

Pharmacological treatment began with the initiation of sertraline, followed by the addition of aripiprazole at a later stage. Initially, sertraline was titrated to 50 mg daily in the morning to address the child’s anxiety and obsessive-compulsive symptoms. However, the dosage was kept at 50 mg as the mother expressed reluctance to increase it due to concerns about side effects. Despite being educated about the medication, the mother initially preferred using supplements. To address her concerns, the child and adolescent psychiatrist introduced valerian supplements, starting with 500 mg of *Valeriana officinalis *extract once daily in the morning during the first appointment. This was maintained for two weeks as a fixed regimen and continued for the following two months as needed (SOS) for significant anxiety. After a week without significant improvement in obsessive-compulsive symptoms, the mother became more accepting of starting sertraline.

With the initiation of sertraline, the obsessive-compulsive symptoms gradually resolved over two months, accompanied by a substantial reduction in anxiety. The child maintained good academic and interpersonal functioning and became more independent, forming a closer bond with his father. Sertraline was continued at 50 mg daily for four months and then reduced to 25 mg for one month.

Two months after discontinuation, the child experienced a mild relapse characterized by repetitive compulsions, such as touching random objects four times, and intrusive ego-dystonic thoughts and images of harming himself and his parents. Sertraline was restarted immediately and titrated to 75 mg over three weeks. Aripiprazole 5 mg once daily at night was also introduced as an augmentative strategy. Symptom remission was achieved after six weeks. This time, sertraline was gradually tapered off after nine months of symptom-free status (75 mg for six months, 50 mg for two months, and 25 mg for one month). Aripiprazole was discontinued after four months of being symptom-free. There was a follow-up appointment every four months after stopping all medications for a year, and the family had direct contact with the service in case of relapse. 

Following the successful treatment, the patient remains in very good condition. He is free of obsessive-compulsive symptoms and exhibits a significantly reduced level of anxiety. He continues to excel academically and socially, with strong interpersonal relationships and increasing independence. The patient is described as emotionally stable, engaged in daily activities, and showing positive growth in self-confidence and resilience.

## Discussion

This case underscores the multifaceted etiology of OCD in children and highlights how the intersection of APA, familial dynamics, and cultural influences shapes symptom presentation and therapeutic responses. The child’s death anxiety and compulsive behaviors were not merely the result of genetic predispositions but were deeply intertwined with the psychosocial environment and caregiving practices within the family.

Genetic and neurobiological factors

APA is associated with de novo mutations and epigenetic changes that disrupt key neurotransmitter pathways, including serotonin and dopamine systems [5,6]. These pathways are critical for regulating emotion and compulsivity, which are core elements of OCD [1]. The resulting structural and functional abnormalities in brain regions like the prefrontal cortex and basal ganglia are well-documented in OCD pathology [1,5,6]. In this case, the child’s genetic vulnerabilities likely set the stage for his obsessive-compulsive behaviors, with death anxiety emerging as a prominent theme due to the interplay between these vulnerabilities and psychosocial stressors.

Family dynamics and death anxiety

The psychological impact of APA often extends beyond genetics to shape familial interactions and emotional development. Older fathers may experience limitations in physical activity and emotional engagement, which can contribute to a perception of emotional unavailability and foster insecure attachment in children [7]. In this case, the father’s expressions of mortality-related regret and fear appeared to amplify the child’s preoccupation with death, intensifying obsessive thoughts and compulsive behaviors. The child’s compulsions, such as cleaning and checking, may reflect an attempt to exert control over his existential fears, which are rooted in the perceived fragility of his father’s health. The fears of losing the caregiver are common in APA families [11].

The family’s avoidance of discussions about mortality played a significant role in the persistence of the child’s symptoms. By not addressing his fears directly, the family might have unintentionally allowed his obsessive thoughts to dominate, preventing the development of adaptive coping mechanisms. This highlights the importance of addressing family communication patterns when treating pediatric OCD, particularly in cases where existential fears are a central feature [12].

Cultural considerations

The mother’s cultural background, characterized by close familial bonds and practices such as co-sleeping, played a dual role [10]. While providing emotional security, these behaviors also seemed to reinforce dependency, limiting the child’s development of independent coping strategies. This enmeshment likely compounded the child’s anxiety and compulsive rituals. For example, her habit of staying in his room at night inadvertently validated his fears, making it more challenging to break the cycle of compulsions. 

A critical component of the intervention was leveraging the spiritual framework of the local Filipino community [10]. By integrating culturally relevant practices, such as exploring the family’s beliefs about death and spirituality, the treatment reframed existential fears within a supportive context. This approach not only aligned with the family’s values but also provided the child with new tools to process his anxiety, transforming potential stressors into sources of stability.

Therapeutic implications

The therapeutic approach in this case was tailored to address the unique interplay of biological, psychosocial, and cultural factors. CBT with ERP is a well-established treatment for pediatric OCD [13] and was central to the intervention, enabling the child to confront his fears about death and gradually reduce his compulsive behaviors. The exposure exercises were designed specifically around his rituals, such as refraining from checking under the bed or rearranging objects, while also addressing the underlying fears tied to his father’s mortality in a supervised environment.

Combined pharmacological intervention with sertraline is effective in OCD by providing critical support by targeting the serotonergic pathways implicated in obsessive-compulsive symptomatology and reducing baseline anxiety, thereby enabling greater engagement in therapy [14]. The addition of valerian supplementation, though less substantiated in rigorous clinical trials [15], was considered to complement anxiety reduction strategies, reflecting an attempt at holistic management of moderate symptoms amidst environmental stressors.

Family-based interventions were another cornerstone of treatment. Sessions focused on fostering father-son interactions, such as shared activities and memory-making, which served to both reduce the child’s anxiety about his father’s health and strengthen their bond. The mother was guided to step back from overinvolvement, promoting the child’s independence while maintaining a supportive role. Psychoeducation played a pivotal role in helping the parents understand the impact of APA, the dynamics of OCD, and the necessity of consistent routines and open communication.

Behavioral strategies were also integral to the intervention. The use of reward systems encouraged the child to sleep alone and gradually face his fears. Symbolic objects, such as the “magic sword” and “monster spray,” provided tangible tools to empower the child in moments of anxiety, blending practical behavioral techniques with imaginative elements tailored to his developmental stage.

## Conclusions

This case underscores the multifaceted impact of APA on the development of OCD in children. While genetic vulnerabilities linked to APA, such as de novo mutations, provide a biological basis for increased risk, psychosocial and familial dynamics significantly influence the clinical presentation. The father’s emotional distancing and preoccupation with mortality, coupled with the mother’s overinvolvement and dependency-reinforcing behaviors, contributed to the child’s compulsions and death-related anxiety. These findings highlight the importance of considering biological predispositions and family dynamics when addressing APA-related challenges.

The therapeutic approach demonstrated the value of a multidisciplinary, culturally sensitive intervention that integrated CBT, family-based strategies, and pharmacological treatment. Fostering open discussions about mortality and strengthening father-son interactions addressed the child’s existential fears while encouraging maternal detachment promoted independence. This case exemplifies the necessity of tailored, holistic treatment plans for children with OCD in APA contexts, emphasizing the need for further research into the complex psychosocial dimensions of APA to guide clinical practice.
